# Supplementary material for: Hydrophobic and Luminescent Polydimethylsiloxane PDMS-Y2O3:Eu3+ Coating for Power Enhancement and UV Protection of Si Solar Cells
Source: Nanomaterials (Basel). 2024 Apr 12;14(8):674. doi: 10.3390/nano14080674 (PMC11054467; doi:10.3390/nano14080674)
Supplement: Supplementary file 1 [file nanomaterials-14-00674-s001.zip › nanomaterials-2931173-supplementary.pdf]

Supporting Information

Hydrophobic and luminescent polydimethylsiloxane PDMS-Y_2O_3:Eu^{3+} coating for power enhancement and UV protection of Si solar cells

Darya Goponenko[1, @], Kamila Zhumanova[1, @], Sabina Shamarova[1], Zhuldyz Yelzhanova[2], Annie Ng[2], and Timur Sh. Atabaev[1, *]

1) Department of Chemistry, School of Sciences and Humanities, Nazarbayev University, Astana 010000, Kazakhstan

2) Department of Electrical and Computer Engineering, School of Engineering and Digital Sciences, Nazarbayev University, Astana, Kazakhstan

@ Equal contribution

* Correspondence: timur.atabaev@nu.edu.kz; Tel.: (+7 7172 70 60 26)

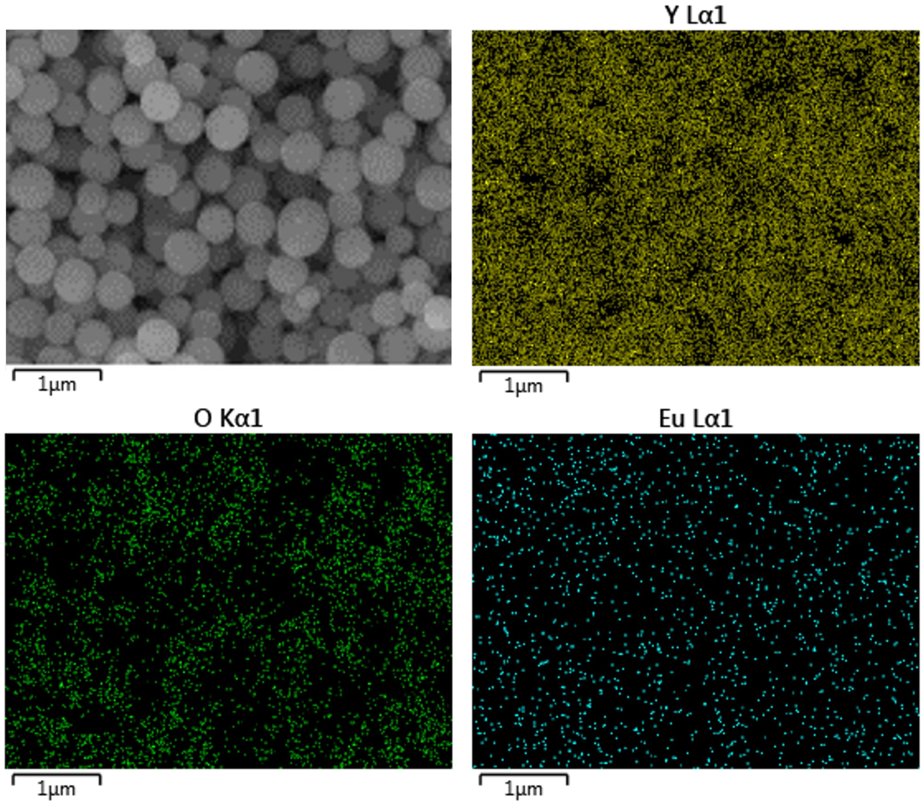

Figure S1. EDS elemental mapping of Y_2O_3:Eu^{3+} particles.

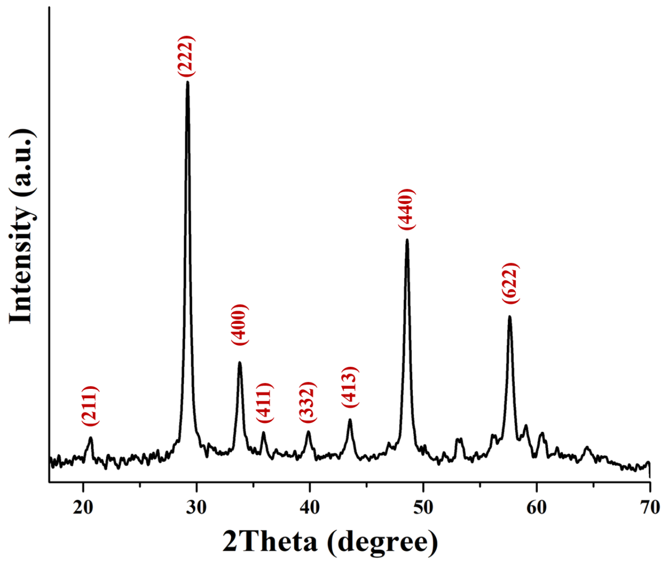

Figure S2. XRD pattern of Y_2O_3:Eu^{3+} particles.

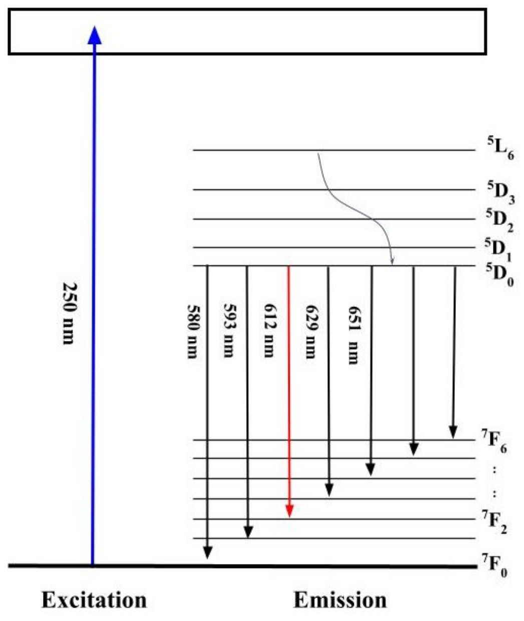

Figure S3. Schematic representation of transitions within Eu^{3+}

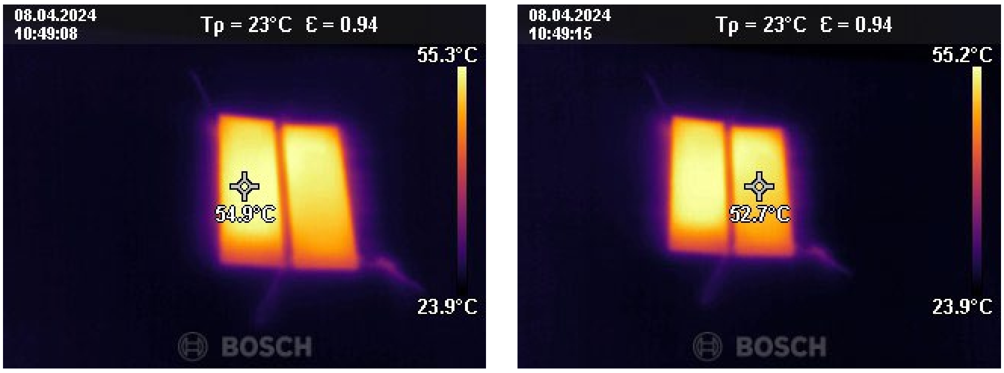

Figure S4. Heating of Si cells within 5 min under simulated solar light illumination. Uncoated Si cell reached ~ 54.9 ºC, while coated Si cell reached ~ 52.7 ºC under the same conditions. Temperature was measured using Bosch GTC 600 C thermal imaging camera.
